# Cholesteryl Ester Transfer Protein Inhibitors and Cardiovascular Outcomes: A Systematic Review and Meta-Analysis

**DOI:** 10.3390/jcdd11050152

**Published:** 2024-05-16

**Authors:** Wajeeh ur Rehman, Merav Yarkoni, Muhammad Abdullah Ilyas, Farwa Athar, Mahnoor Javaid, Muhammad Ehsan, Muhammad Talha Khalid, Ahmed Pasha, Abdelhamid Ben Selma, Alon Yarkoni, Keyoor Patel, Mouhamed Amr Sabouni, Afzal ur Rehman

**Affiliations:** 1 Heart and Vascular Institute, United Health Services, Johnson City, NY 13790, USA; ahmed.pasha@nyuhs.org (A.P.); alonyarkoni@gmail.com (A.Y.); keyoor.patel@nyuhs.org (K.P.); afzal.rehman@nyuhs.org (A.u.R.); 2 Department of Medicine, King Edward Medical University, Lahore 54000, Pakistan; muhammabdullahilyas@kemu.edu.pk (M.A.I.); farwaathar@kemu.edu.pk (F.A.); m.ehsanqadri@gmail.com (M.E.); 3 School of Medicine, CMH Lahore Medical College, Lahore 54000, Pakistan; mahnoorjavaid713@gmail.com; 4 Department of Medicine, United Health Services, Johnson City, NY 13790, USA; muhammad.khalid@nyuhs.org (M.T.K.); abdelhamid.benselma@nyuhs.org (A.B.S.); 5 Cardiovascular Disease, University of Alabama at Birmingham, Birmingham, AL 35294, USA; msabouni@uabmc.edu

**Keywords:** atherosclerosis, cholesterol ester transfer protein, CETP inhibitors, HDL-C lipoproteins, LDL-C lipoproteins, anacetrapib, dalceprapib, evacetrapib, obicetrapib, torcetrapib

## Abstract

Background: Atherosclerosis is a multi-factorial disease, and low-density lipoprotein cholesterol (LDL-C) is a critical risk factor in developing atherosclerotic cardiovascular disease (ASCVD). Cholesteryl-ester transfer-protein (CETP), synthesized by the liver, regulates LDL-C and high-density lipoprotein cholesterol (HDL-C) through the bidirectional transfer of lipids. The novelty of CETP inhibitors (CETPis) has granted new focus towards increasing HDL-C, besides lowering LDL-C strategies. To date, five CETPis that are projected to improve lipid profiles, torcetrapib, dalcetrapib, evacetrapib, anacetrapib, and obicetrapib, have reached late-stage clinical development for ASCVD risk reduction. Early trials failed to reduce atherosclerotic cardiovascular occurrences. Given the advent of some recent large-scale clinical trials (ACCELERATE, HPS3/TIMI55-REVEAL Collaborative Group), conducting a meta-analysis is essential to investigate CETPis’ efficacy. Methods: We conducted a thorough search of randomized controlled trials (RCTs) that commenced between 2003 and 2023; CETPi versus placebo studies with a ≥6-month follow-up and defined outcomes were eligible. Primary outcomes: major adverse cardiovascular events (MACEs), cardiovascular disease (CVD)-related mortality, all-cause mortality. Secondary outcomes: stroke, revascularization, hospitalization due to acute coronary syndrome, myocardial infarction (MI). Results: Nine RCTs revealed that the use of a CETPi significantly reduced CVD-related mortality (RR = 0.89; 95% CI: 0.81–0.98; *p* = 0.02; I2 = 0%); the same studies also reduced the risk of MI (RR = 0.92; 95% CI: 0.86–0.98; *p* = 0.01; I2 = 0%), which was primarily attributed to anacetrapib. The use of a CETPi did not reduce the likelihood any other outcomes. Conclusions: Our meta-analysis shows, for the first time, that CETPis are associated with reduced CVD-related mortality and MI.

## 1. Introduction

It is now well established that low-density lipoprotein cholesterol (LDL-C) and apolipoprotein B (ApoB) are the two main risk factors that cause atherosclerotic cardiovascular disease (ASCVD). The evidence from numerous studies indicates that reducing the plasma concentration of LDL-C reduces the risk of ASCVD [1]. In recent decades, the foundations of lipid-lowering therapies were largely based on statins, but also on ezetimibe, proprotein convertase subtilisin–kexin Type 9 (PCSK9) inhibitors, and more recently, bempedoic acid [2], and these therapies improved the outcomes by roughly 30%. Since then, clinicians have discovered that a meaningful proportion of cardiovascular events cannot be prevented solely by lowering LDL-C. Since atherosclerosis is a multi-factorial disease, a new focus has been placed on developing strategies to increase the amount of high-density lipoprotein cholesterol (HDL-C) [3], giving rise to a new era of cholesteryl ester transfer protein (CEPT) inhibitors. In contrast to LDL, HDL is prominent for its role in reverse cholesterol transport, removing cholesterol from peripheral tissues into the liver, and providing anti-inflammatory, anti-thrombotic, anti-oxidative, and anti-atherogenic properties [4,5].

CETP is a glycoprotein synthesized by the liver; it has a special banana shape that enables it to bind both cholesteryl esters and triglycerides [6], and facilitates the bidirectional transfer of these molecules between all plasma lipoprotein particles. Most plasma cholesteryl esters are found within HDL particles, while triglycerides are mainly found within very low-density lipoprotein (VLDL) particles and chylomicrons. CETP activity results in a net mass transfer of cholesteryl esters from cholesteryl ester-rich HDL particles to VLDL and LDL. In a similar fashion, there is a net mass transfer of triglycerides from triglyceride-rich VLDL particles and chylomicrons to LDL and HDL particles [4,7,8,9,10]. This dual mechanism has a direct impact on the levels of both LDL-C and HDL-C in the plasma [7,8,9,10]. Inhibiting CETP reduces these exchanges resulting in increased concentrations of cholesterol in HDL and decreased concentrations of cholesterol in apolipoprotein (Apo) B-containing particles, i.e., VLDL and LDL [4]. Interestingly, CETP is present and active in all primates, rabbits, and hamsters, but is lacking in the plasma of most other species [11]; this phenomenon directly influences the development of resistance to atherosclerosis in opposing species. For instance, rabbits on a high-cholesterol diet develop atherosclerosis, while rodents which lack CETP are naturally resistant to the development of atherosclerosis [12].

In human studies, the initial interest in pharmacological CETP inhibition was instigated by the discovery that Cept gene mutations led to significantly elevated HDL-C levels and decreased LDL-C levels in multiple families [13,14,15,16,17,18]. Several single-nucleotide polymorphisms (SNPs) in the Cept gene were associated with decreased CETP activity and with a lower ASCVD risk (reviewed in [12]). It was initially hypothesized that the reduced ASCVD risk that is associated with mutated Cept genes is driven by mechanisms that lower LDL-C and other atherogenic lipoproteins rather than those that increase HDL-C levels [12,19]. However, prospective epidemiological studies have clearly shown that a low HDL-C level is a strong and independent risk factor associated with the development of cardiovascular disease [20,21,22,23].

To date, five CETP inhibitors including torcetrapib, dalcetrapib, evacetrapib, anacetrapib, and, most recently, obicetrapib have reached late-stage clinical development for ASCVD risk reduction (reviewed in [24]). It has been observed that all CETP inhibitors have a significantly positive effect on apolipoprotein A-I and HDL-C concentrations. These drugs are expected to decrease the mobility of cholesterol esters from HDL to ApoB-containing lipoproteins, resulting in an increase in HDL-C levels. The majority of these medications also reduce the levels of LDL-C and ApoB, along with the concentration of the lipoprotein A which is atherogenic. Therefore, the impact of CETP inhibitors on the lipid profile appears to be advantageous in relation to the majority of lipid fractions [25].

The development of first-generation (torcetrapib and calcertrapid) and second-generation CEPT inhibitors (anacetrapib and evacetrapid) subsequently enhanced the efficacy of this treatment by not only elevating HDL-C levels, but also reducing LDL-C levels. In numerous trials, the main hypothesis was that an increase in HDL-C would lead to a decrease in MACEs. Nonetheless, the Evaluation of the Effects of Anacetrapib Through Lipid Modification (REVEAL) trial demonstrated a significant reduction in MACEs that was directly correlated with reductions in non-HDL-C molecules [12,19,26,27,28].

Previous clinical trials of the above four CETP inhibitors lacked guaranteed results and raised safety concerns. Nevertheless, the genetic studies showed that CETP deficiency is an independent ASCVD risk modulator [17]. Therefore, better pharmacological CEPT inhibitors are needed to reduce ASCVD. 

Randomized controlled trials (RCTs) of drugs that increase HDL-C, such as niacin and fibrates, have not supported the hypothesis for the use of HDL-C for ASCVD risk reduction [4]. As additional large clinical trials of CETP inhibitors have been published, specifically the ACCELERATE trial-2020 and HPS3/TIMI55-REVEAL Collaborative Group-2022, it is necessary to consolidate and strengthen the existing evidence regarding the effectiveness of CETP inhibitors [27,29] for ASCVD risk reduction. Several studies on CETP regulation have been conducted in the past decade [30]. Consequently, we conducted a meta-analysis of RCTs to evaluate the impact of CETP inhibitors on cardiovascular outcomes, which is currently lacking positive results. We show, for the first time, the significant association between the first- and second-generation CETP class inhibitors and reducing the risk of MACE, all-cause mortality, cardiovascular disease (CVD) mortality, MI, and revascularization. We also present herein a broad overview of each of these drugs and discuss their positive and negative outcomes before finally introducing the next generation of CETP class inhibitors. Knowing the clinical effects of these inhibitors may help us to develop more individualized and focused treatment plans for those at risk of CVD, which would ultimately enhance patient outcomes and quality of life.

## 2. Materials and Methods

The meta-analysis we conducted adhered to the guidelines outlined in the Preferred Reporting Items for Systematic Reviews and Meta-Analyses (PRISMA) [31].

### 2.1. Search Strategy

A systematic search was performed on Pubmed, Embase, MEDLINE, and the Cochrane Library, covering the period from the beginning of these databases in 2003 until 27 October 2023. The objective was to identify RCTs that evaluated the effectiveness and safety of CETP inhibitors. The search strategies employed in this study involved the use of specific drug names. These drug names included anacetrapib (MK-0859), evacetrapib (LY2484595), obicetrapib (TA-8995), and dalcetrapib (JTT-705). The search strategy is mentioned in Supplementary Table S1. In addition, we conducted a thorough examination of the Reference lists of the trials included in our study and relevant reviews in order to identify any RCTs that were not identified through our electronic search. Finally, a search was conducted on ClinicalTrials.gov to identify RCTs that have not yet been published.

### 2.2. Inclusion and Exclusion Criteria

During the electronic search process, two reviewers conducted a thorough examination of the titles and abstracts of all publications that were identified. This examination was carried out independently by each reviewer. Two independent reviewers conducted a full-text review of publications that were potentially eligible. Disagreements that arose during this stage were resolved either through the establishment of a consensus or, if deemed necessary, by engaging a third reviewer. Publications were considered eligible if they met the following criteria: (1) data were analyzed from RCTs that compared a CETP inhibitor to a placebo; (2) the participants were 18 years and older; (3) the trials enrolled at least 100 participants; (4) the follow-up duration was at least 6 months; and (5) the study reported at least one of the specified primary or secondary outcomes. Publications were excluded if they featured torcetrapib as the CETP inhibitor, and if the adopted study designs were in the format of a review, editorial, or a letter to the editor. When multiple publications arose from the same trial, priority was given to the publication that provided the most extensive and comprehensive reporting. It is important to note that conference abstracts were not included in our study if a corresponding published manuscript was found.

### 2.3. Data Extraction

Two reviewers, working independently, collected data from RCTs that satisfied the criteria for inclusion. Discrepancies were resolved either through consensus among the involved parties or by involving a third reviewer. The study characteristics that were extracted included the year of publication, drug regimen used, and the number of participants in each arm. The baseline characteristics of the patients were assessed, which included their age, body mass index, sex, usage of statins, and cardiovascular risk factors like prevalence of hypertension and diabetes. Additionally, initial measurements of HDL-C and LDL-C were documented.

Our primary outcome of interest was MACEs, CVD mortality, and all-cause mortality. The secondary outcomes of our study were stroke, revascularization, hospitalization due to acute coronary syndrome, and myocardial infarction (MI). 

### 2.4. Quality Assessment and Statistical Analysis

The quality assessment of the study was conducted by an independent researcher (referred to as XY) using the Risk of Bias Tool 2 (ROB 2.0) [32]. The results of this assessment can be found in Supplementary Figure S1. The forest plots and statistical analysis were performed using Review Manager 5.4. The pooled effect size was computed by employing forest plots with a random effects model. To evaluate publication bias, we employed a funnel plot (Supplementary Figure S2). A *p*-value less than 0.05 was considered statistically significant. We performed sub-group analyses on the basis of the type of drug used, for each outcome, in order to keep the heterogeneity in check and to make our data more clinically useful. 

## 3. Results

### 3.1. Literature Search Results

**Figure 1:**
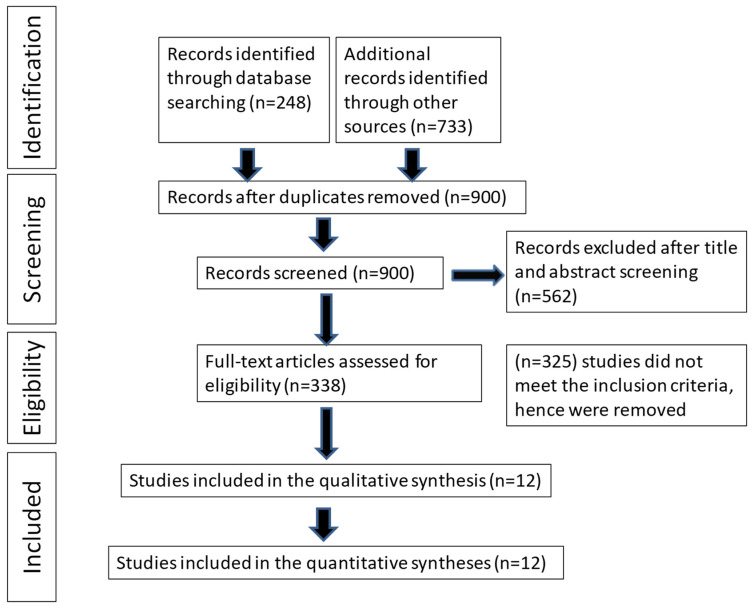
PRISMA flow diagram.

Figure 1 depicts the process of selecting the studies. The initial search yielded 981 studies, with 900 duplicate records being identified and subsequently eliminated. An additional 562 studies were excluded based on irrelevant titles and abstracts. The remaining 338 studies underwent further assessment to ensure their relevance to the subject. Following this, 325 studies were excluded because they did not meet the eligibility criteria. Consequently, the final selection comprised 12 RCTs that were used for the meta-analysis.

### 3.2. Baseline Characteristics 

Table 1 shows the baseline characteristics of the studies included in this meta-analysis. The study encompassed a total of 104,799 participants. 

### 3.3. Results of the Meta Analysis

#### 3.3.1. Major Adverse Cardiovascular Events (MACEs)

**Figure 2:**
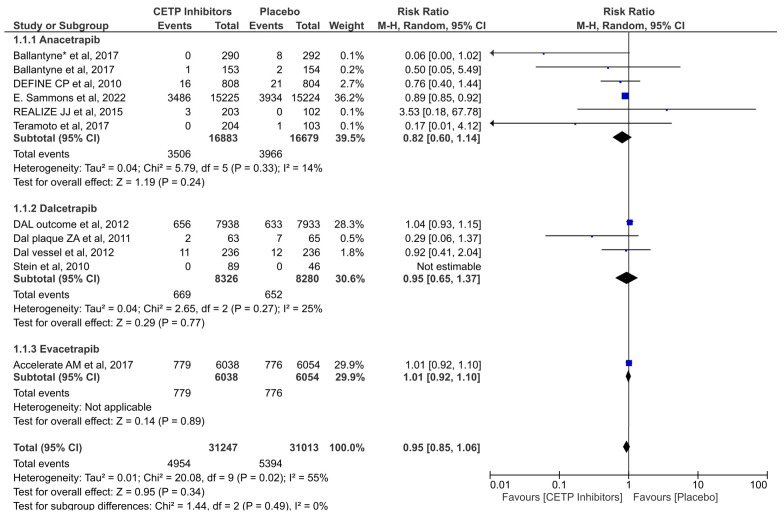
Forest plot of MACEs outcome [10,27,29,33,34,35,36,37,38,39,40] *. The values of each study (represented by black diamonds), in which the size is determined by 95% CI; the effect size of each individual study in the meta-analysis (represented by blue squares).

Data from 11 of the RCTs were pooled to evaluate the effect of the CETP inhibitors in reducing the risk of MACEs. It was revealed that there was no significant decrease in the risk of MACE in the group treated with the CETB inhibitors versus the placebo group (RR = 0.95; 95% CI: 0.85–1.06; *p* = 0.34; I2 = 55%) (Figure 2). There was no significant difference between the three subgroups with regard to this outcome (*p* = 0.49). 

#### 3.3.2. Cardiovascular Disease (CVD) Mortality

**Figure 3:**
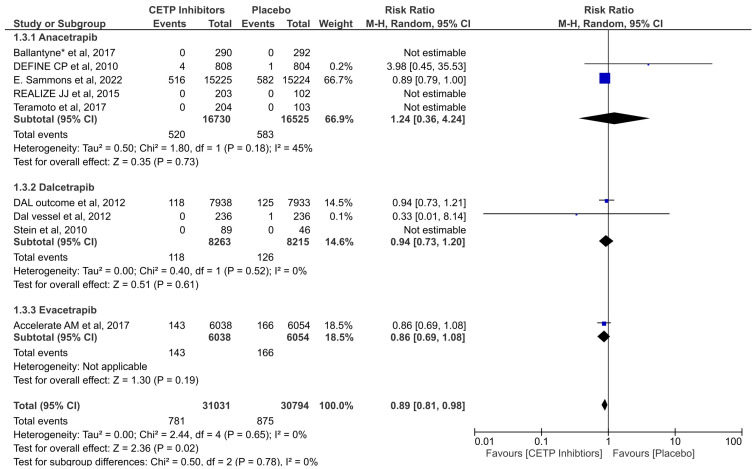
Forest plot of CVD mortality outcome [10,27,29,33,35,36,37,38,40] *. The values of each study (represented by black diamonds), in which the size is determined by 95% CI; the effect size of each individual study in the meta-analysis (represented by blue squares).

The findings of nine of the studies were combined to evaluate the impact of CETP inhibitors on CVD mortality when compared to a placebo group. The significant results favored the CETP inhibitors, proving their success in decreasing the risk of CVD mortality (RR = 0.89; 95% CI: 0.81–0.98; *p* = 0.02; I2 = 0%) (Figure 3). There was no significant difference between the three subgroups in terms of this outcome as well (*p* = 0.78).

#### 3.3.3. All-Cause Mortality

**Figure 4:**
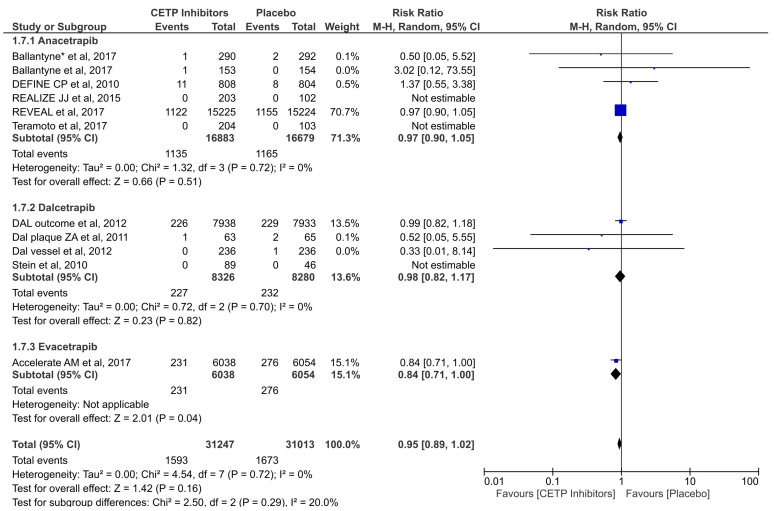
Forest plot of the all-cause mortality outcome [10,27,29,33,34,35,36,37,38,39,40] *. The values of each study (represented by black diamonds), in which the size is determined by 95% CI; the effect size of each individual study in the meta-analysis (represented by blue squares).

Eleven studies assessed the effectiveness of CETP inhibitors in reducing the risk of all-cause mortality. The results suggested the CETP inhibitors were not significantly associated with reducing the risk of all-cause mortality compared to the placebo group (RR = 0.95; 95% CI: 0.89–1.02; *p* = 0.16; I2 = 0%) (Figure 4). Testing for subgroup differences was not statistically significant (*p* = 0.29).

#### 3.3.4. Myocardial Infarction (MI)

**Figure 5:**
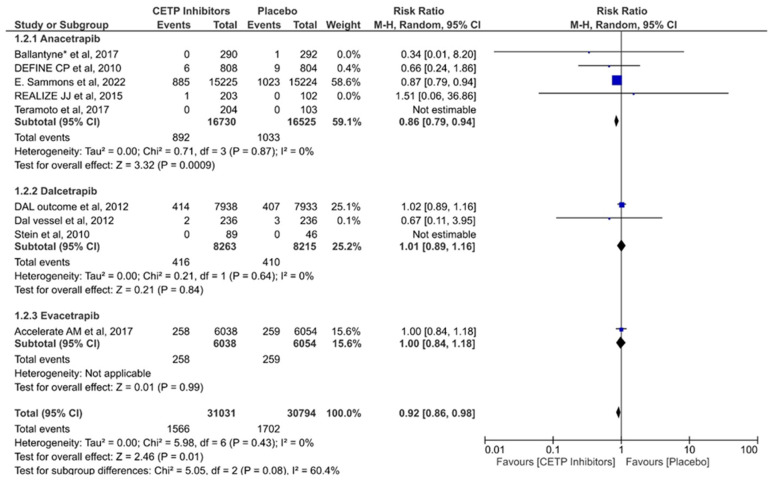
Forest plot of the myocardial infarction outcome [10,27,29,33,35,36,37,38,40] *. The values of each study (represented by black diamonds), in which the size is determined by 95% CI; the effect size of each individual study in the meta-analysis (represented by blue squares).

Nine studies assessed the effectiveness of CETP inhibitors in reducing the risk of MI. The results suggested the CETP inhibitors were significantly associated with reducing the risk of MI compared to the placebo group (RR = 0.92; 95% CI: 0.86–98; *p* = 0.01; I2 = 0%) (Figure 5). Testing for subgroup differences yielded an interesting picture for this outcome (*p* = 0.08). For the Anacetrapib subgroup, the use of the drug significantly reduced the risk of MI (RR = 0.86; CI: 0.79–0.94; *p* = 0.0009; I2 = 0%). However, the analyses of Dalcetrapib (RR = 1.01; CI: 0.89–1.16; *p* = 0.84; I2= 0%) and Evacetrapib (RR = 1.00; CI: 0.84–1.18; *p* = 0.99) did not show any significantly favorable impacts of the drug compared to the placebo. 

#### 3.3.5. Stroke

The results of nine of the studies were aggregated to assess the influence of CETP inhibitors on the risk of stroke in comparison to a placebo group. The pooled analysis revealed no significant differences in reducing the risk of stroke between both groups (RR = 0.97; 95% CI: 0.87–1.08; *p* = 0.61; I2 = 6%) (Supplementary Figure S3). There was no reportable difference between the different subgroups (*p* = 0.20) [10,27,29,33,35,36,37,38,40].

#### 3.3.6. Hospitalization Due to Acute Coronary Syndrome

The findings of eight of the studies were pooled to assess the influence of CETP inhibitors on hospitalization due to acute coronary syndrome in comparison to a placebo group. The pooled analysis revealed no significant differences in reducing the risk of said outcome between both groups (RR = 0.93; 95% CI: 0.68–1.28; *p* = 0.66; I2 = 31%) (Supplementary Figure S4). Running the subgroup analyses for this outcome revealed no significant difference (*p* = 0.40) [10,27,29,33,35,36,37,38,40].

#### 3.3.7. Revascularization 

Data from nine of the RCTs were pooled to evaluate the effect of CETP inhibitors in reducing the risk of revascularization. It was revealed that there was no significant decrease in the risk of revascularization in the group of CETP inhibitors versus the placebo group (RR = 0.93; 95% CI: 0.81–1.07; *p* = 0.30; I2 = 74%) (Supplementary Figure S5). There was also no significant difference between the three subgroups for this outcome (*p* = 0.53) [10,27,29,33,35,36,37,38,40]. 

### 3.4. Results of Previous RCTs

Table 2 lists all of the RCTs with lipid outcomes for high-risk CVS patients, the endpoints, side and toxic effects, termination time, and reasons for termination. In the Investigation of Lipid Level Management to Understand its Impact in Atherosclerotic Events (ILLUMINATE) trial, 60 mg of torcetrapib taken once daily increased the incidence of death and CVD events which led to premature termination of the trial [41]. After a median of 550 days, in ASCVD patients receiving torcetrapib, in a randomized, double-blind fashion, the drug increased HDL-C by an average of 72% and lowered LDL-C by 25% (Table 2, [41]). 

In the dalcetrapib on cardiovascular mortality and morbidity in clinically stable patients with a recent acute coronary syndrome (dal-OUTCOMES) trial, no significant association was observed between higher HDL-C levels (40% increase) and the risk of MACEs (a composite of death from coronary heart disease, non-fatal MI, ischaemic stroke, unstable angina, or cardiac arrest with resuscitation); treatment with 600 mg of dalcetrapib had a negligible effect on LDL-C and apoB levels (Table 2, [37]). This trial was terminated after reaching 71% of the projected total number of events at 34 months [37]. 

In contrast to dalcetrapib, the assessment of the clinical effects of CETP inhibition with evacetrapib in patients at a high-risk for vascular outcomes (ACCELERATE) [29] trial did lower LDL-C and apoB levels significantly but modestly (by 22% and 14%, respectively), and simultaneously produced larger increases in HDL-C concentrations (by 95%) in early clinical trials [42]. However, 130 mg of dalcetrapib was ineffective in reducing the primary endpoints (composite of death from cardiovascular causes, MI, stroke, coronary revascularization, or hospitalization for unstable angina). Like the Dal-OUTCOMES trial, the ACCELERATE trial was terminated prematurely at a mean of 26 months after 82% of the planned primary endpoint events because of a lack of efficacy (Table 2, [29]). 

The randomized evaluation of the effects of anacetrapib through lipid modification (REVEAL) was the largest randomized, double-blind, placebo-controlled trial for CETP inhibition to date, with a mean of a 4-year follow-up (Table 2, [26]). The primary endpoint was defined as the first major coronary event (a composite of coronary death, MI, or coronary revascularization). Secondary outcomes were major atherosclerotic events (a composite of coronary death, MI, or ischaemic stroke), ischaemic stroke, and major vascular events (a composite of major coronary events or ischaemic stroke). Treatment with 100 mg anacetrapib did not significantly decrease death rates from CVD causes, all non-CVD causes, or all-cause mortality compared to the placebo. Nevertheless, lipid profiles improved significantly with a 104% increase in HDL-C and a 23% decrease in LDL-C (Table 2, [26]).

Obicetrapib (also known as TA-8995) at a low dose of 5 mg increased HDL-C by 157% and decreased LDL-C by 45% and 63% in the TULIP and ROSE2 trials, respectively, after 12 weeks (Table 2, [43,44]).

**Table 2:** Lipid outcomes at longest follow-up in randomized controlled trials with use of CETP inhibitors.

Drug	Dose (mg)	Trial	Patients	HDL-C (mmol/L)% Change from Baseline	LDL-C (mmol/L)% Change from Baseline	Endpoints	Side Effects (Increases in)	Target Toxic Effects, Other	Termination, Reason	Reference
Torcetrapib + atorvastatin	90		High CVS risk	+40.2	−18.9	MACE, NS	SBP, DBP in some patients	NS	8 weeks, NS	[45]
Torcetrapib + atorvastatin	60	ILLUMINATE	High CVS risk	+72.1	−24.9	Time to 1st MACE	SBP, sodium, bicarbonate, aldosterone, decreased potassium	Risk of CVS events, death from any cause	12 months, incr. risk of death and CVS events	[41]
Dalcetrapib	600	dal-OUTCOMES	Acute coronary syndrome	+40	Minimal	ASCVD death, NS	SBP, C-reactive protein	Improved endothelial function	31 months, futility	[37]
Dalcetrapib	900		Mild hyperlipidemia	+34	−7	Phase II	Phase II	None	4 weeks	[46]
Dalcetrapib	600	dal-VESSEL	CHD	+31	NA	%FMD, ABPM	NA	NA	36 weeks	[38]
Dalcetrapib	100	REALIZE	Hyper-cholesterolaemia, high CVS risk	NA	−40	%LDL-C	NS	Increased CVS events	52 weeks, additional F/U 12 weeks	[36]
Anacetrapib	100	HPS3/TIMI55– REVEAL	ASCVD	+104	−23.4	MACEs Decreased ASCVD, plasma non-HDL-C, new onset diabetes	SBP, DBP	No safety issues	4 years, not approved due to high lipophicity and accumulation in adipose tissue	[26]
Anacetrapib	100	DEFINE	CHD	+138.1	−39.8	CVS events, deaths	Acceptable	NS	76 weeks, did not result in adverse CVS effects	[10]
Anacetrapib	100		Hyper- cholesterolemia	+118	−37	%HDL-C, %LDL-C, safety profile of anacetrapib	NS	NS	24 weeks	[33]
Anacetrapib	100		Dylipidemia, history of CHD	+149	−38	%LDL-C, safety profile of anacetrapib	None	None	24 weeks, F/U 52 weeks	
Evacetrapib	130	ACCELERATE	High CVS risk	+94.6	−22.3	MACE, NS	None	None	26 months, lack of efficacy	[29]
Evacetrapib	100		Dyslipidemia	+128.8	−35.9	NS	None	None	12 weeks, Lack of efficacy	[47]
Evacetrapib	100	ACCELERATE	Diabetes mellitus	+131	−32	Time to 1st MACE	NS	NS	26 months	[48]
Obicetrapib	5	TULIP	Mild dyslipidemia	+157.1	−45.3	Phase II	None	None	12 weeks	[43]
Obicetrapib +ezetimibe	10	ROSE2	Patients with elevated LDL-C	NA	−63	Phase II	Lipid concentrations, safety, and tolerability.	None	12 weeks	[44]

ABPM: ambulatory blood pressure monitoring; ACCELERATE: Assessment of Clinical Effects of Cholesteryl Ester Transfer Protein Inhibition with Evacetrapib in Patients at a High-Risk for Vascular Outcomes; ASCVD: Atherosclerotic cardiovascular disease; CHD: coronary heart disease; CVS, cardiovascular; dal-OUTCOMES: Dalcetrapib on Cardiovascular Mortality and Morbidity in Clinically Stable Patients with a Recent Acute Coronary Syndrome; dal-VESSEL: A Study Assessing the Effect of Dalcetrapib on Vascular Function in Patients With Coronary Heart Disease (CHD) or CHD-Risk Equivalent Patients; DBP: diastolic blood pressure; DEFINE: Details of the Determining the Efficacy and Tolerability of CETP Inhibition with Anacetrapib; FMD: flow-mediated dilatation; HDL-C: high-density lipoprotein cholesterol; ILLUMINATE: Investigation of Lipid Level Management to Understand its Impact in Atherosclerotic Events; LDL-C: low-density lipoprotein cholesterol; MACEs: major adverse cardiovascular events; NA: not applicable; NS: not significant; REALIZE: Anacetrapib as lipid-modifying therapy in patients with heterozygous familial hypercholesterolaemia; REVEAL: Randomized Evaluation of the Effects of Anacetrapib through Lipid Modification.

## 4. Discussion

In this meta-analysis we reveal, for the first time, that CETP inhibitors significantly reduce CVD mortality and MI risk outcomes in ASCVD patients. To our knowledge, no previous reports have established these results with first- and second-generation and newer CETP inhibitors. Our data also demonstrate that there are no significant differences for the outcomes of MACEs, all-cause mortality, stroke, or hospitalization due to acute coronary syndrome, and revascularization between CTEP inhibitors and placebo groups. Our subgroup analysis between the different CEPT inhibitor agents further validated these findings. The lack of change in the stroke or hospitalization outcomes due to acute coronary syndrome may be due to the fact that we could not clearly separate ischemic (thrombotic) strokes from hemorrhagic (hypertensive) strokes in which lipid profiles are not likely implicated. 

A number of previous meta-analyses were conducted to evaluate the impact of CETP inhibitors on cardiovascular outcomes and mortality [49,50,51], but were inconsistent or incomplete compared to our results. In a 2014 study by Keene et al., neither CETP inhibitors, nor niacin or fibrates, all of which effectively raise HDL-C levels, reduced all-cause mortality, coronary heart disease mortality, MI, or stroke in patients treated with statins [49], the mainstay of lipid-lowering therapy. Meanwhile, our study successfully showed a decrease in the risk of MI and CVD mortality, regardless of patient treatment regime. A second study conducted in 2015 by Verodia et al. failed to show lower rates of CVD mortality with CETP inhibitors or niacin, irrespective of increases in HDL-C intensity [50]. The authors demonstrated that there were significant benefits of using niacin for MI and coronary revascularization, but this was not the case for CETP inhibitors despite the higher occurrence of diabetes in the treated patients [50]. Finally, the most recent meta-analysis presented by Taheri et al. in 2020 neglected to show, like us, the significant impact of CETP inhibitors in reducing the risk of MACE or all-cause mortality [51]. The authors concluded that there is a trend towards small reductions in nonfatal MI and cardiovascular death, which we validated in our study. 

Three of the early CETP inhibitors, namely torcetrapib, dalcetrapib, and evacetrapib, were unsuccessful in demonstrating a reduced risk of ASCVD for a variety of reasons in multiple large Phase III clinical trials. The use of the first CETP inhibitor, torcetrapib, assessed in the ILLUMINATE trial in 2007, was terminated after causing more death and CVD events [41]; at that time, the mechanisms were unknown. Later, it was revealed that torcetrapib had structure-related off-target effects causing increased blood pressure, as well as augmented aldosterone, cortisol, and endothelin-1 levels, in addition to profound changes in serum potassium and bicarbonate [52,53]. Torcetrapib also impaired endothelial function in hypertension patients [52,53] and significantly increased systolic and diastolic blood pressures [45].

None of the CETP inhibitors developed after torcetrapib had similar off-target side effects, and all have demonstrated favorable safety profiles [26,27,29,37,44,54]. In 2012, the dalcetrapib study evaluated the effect of cardiovascular mortality and morbidity in patients with acute coronary syndrome in the randomized, double-blind, placebo-controlled (Dal-OUTCOMES) trial [37]. Despite increasing HDL-C levels, dalcetrapib, administered at a dose of 600 mg, was ineffective in reducing cardiovascular morbidity and mortality and this trial was terminated early. Two years later, in 2017, the next CETP inhibitor reached phase III clinical development in the ACCELERATE trial [29]. It is likely that, due to its early termination, the ACCELERATE study was too short to detect a significant reduction in MACE [42]. Yet with minimal side effects to this medication, an apparently significant reduction in total mortality was observed.

In the same year, another effective CETP inhibitor for cardiovascular outcomes, anacetrapib, was developed in the REVEAL trial. It showed a significantly lower incidence of major coronary events among patients with ASCVD who were receiving intensive statin therapy [26]. The exceptionally long follow-up was a direct result of anacetrapib accumulating in adipose tissue and lengthening the terminal half-life of the drug [55,56,57]. Overall, there were no significant safety issues. Patients were followed up after the end of the treatment phase for a median period of 2.3 years to investigate longer-term safety and efficacy, which demonstrated a further 20% reduction in coronary events [27]. Interestingly, compared with the other CETP inhibitors, new-onset diabetes mellitus occurred less frequently in the anacetrapib-treated patients compared to the placebo group. Additionally, significant effects were seen on the rates of CVD death after anacetrapib treatment. In summary, the absolute reduction in major coronary events after 4 years doubled during the post-trial follow-up of more than 2 years. The combined overall proportional reduction in major coronary events over the full 6.3 years median follow-up was 12% [27]. The lower number of events correlated with reduced ApoB particles, and was not associated with elevated HDL-C [12,19,26,27]. The drug was not approved [58] due to its accumulation in adipose tissue and its high lipophicity [59]. In conclusion, anacetrapib is the most lipophilic CETP inhibitor and has an exceptionally long elimination half-life of years rather than hours as seen with the other CETP inhibitors, most likely due to its prolonged and higher accumulation in adipose tissue than in plasma [59,60].

During the development of CETP inhibitors for reducing the risk of ASCVD, significant thought was directed towards raising HDL-C concentrations. However, as evidenced from recent studies in animal models and human cohorts, there is a new focus on lowering the concentrations of LDL-C, non-HDL-C, and ApoB [61,62,63]. The most up-to-date CETP inhibitor to reach late-phase clinical development is obicetrapib. It was shown to robustly reduce LDL-C, ApoB, and other atherogenic lipoproteins while increasing HDL-C particles, ApoA1, and ApoE [44,54,63]. 

Since the discontinuation of anacetrapib, newer CETP inhibitors are currently undergoing phase III clinical development; in addition to obicetrapib (TA-8995) described below, these also include CKD-508 and MK-8262, all of which are beyond the scope of this meta-analysis [4,44,64,65,66,67,68].

Obicetrapib is an oral, once-daily, low-dose, safe, and tolerable CETP inhibitor under development for the treatment of dyslipidemia, CVD risk, and Alzheimer’s disease. It is emerging as a first-in-class CETP inhibitor available for clinical use, and may be a promising agent for the treatment of ASCVD. Early phase I and II trials with obicetrapib showed very promising results with minimal doses of 5 mg per day, and the drug was proven to lower atherogenic lipoproteins while raising HDL-C particles [43,44,54,61,69,70]. The TULIP [43], ROSE [61], ROSE2 [44] and OCEAN [71] trials, performed in participants with dyslipidaemia, demonstrated significant reductions in LDL-C and apoB, whereas HDL-C was increased considerably after 12 or 8 weeks of medication. There were no side effects of high blood pressure or changes in aldosterone, sodium, bicarbonate, high-sensitivity C-reactive protein, or endothelin-I levels. No other serious adverse or toxic effects were reported in these early phase trials [43,54]. There are currently three ongoing phase III trials with obicetrapib [66,67,68], one of which is on CVD outcomes. The BROADWAY, with over 2500 patients with established ASCVD who require additional LDL-C-lowering [67,72]; the BROOKLYN trial with 354 participants across ten countries in North America, Europe, and Africa investigating Heterozygous Familial Hypercholesterolemia [66,72]; and the PREVAIL, which is targeting the enrollment of 9000 participants for the assessment of cardiovascular outcomes [68]. PREVAIL explores the potential of obicetrapib to reduce MACEs incidence (cardiovascular death, non-fatal MI, non-fatal stroke, and non-elective coronary revascularization) in patients with a history of ASCVD with poor LDL-C control regardless of statin therapy. Results from PREVAIL are expected in 2026.

The primary mechanism and outcome of CETP inhibition is the rate of the reduction in cholesteryl ester transfer from HDL into triglyceride-rich lipoproteins [73], thereby increasing plasma HDL-C levels. This results in there being a higher cholesterol content in HDL particles, making them larger slow-metabolizing molecules. HDL has cardio protective properties through a range of pathways, encompassing antiapoptotic, antithrombotic, anti-inflammatory, and antioxidative actions [74]. Additionally CETP inhibitors reduce cholesterol content in ApoB molecules, including VLDL, LDL, chylomicrons, and their remnants [75]. CETP inhibitors enhance cholesterol efflux capacity, which is the initial stage in the process of reverse cholesterol transport [29,43,76], suggesting that HDL-C helps move cholesteryl esters from atherosclerotic lesions to steroid-making organs like the liver, and thereby reduces the buildup of plaque [77]. Taken together, it is important to keep in mind that CETP can either be pro- or antiatherogenic, depending on the metabolic setting. 

Investigating CETP inhibitors in clinical settings could pave the way for new treatment approaches to lessen the burden of CVD events and related mortality. If successful, CETP inhibitors might become a useful supplement to current treatment plans, giving medical practitioners one more option to monitor and avoid CVD problems. Knowing the clinical effects of these inhibitors may help us to develop more individualized and focused treatment plans for those at risk of CVD, which would ultimately enhance patient outcomes and quality of life.

Novel research on CETP inhibitors presents exciting opportunities to improve our knowledge of cardiovascular health, particularly further investigations of the detailed mechanisms by which CETP inhibitors affect mortality and CVD outcomes. Thorough exploration can also aid in the discovery of putative biomarkers or patient traits that might indicate a positive response to CETP inhibitors. To optimize the use of these inhibitors in a variety of patient populations and to establish evidence-based therapeutic guidelines, further research into the long-term effects and safety profiles of these drugs is essential. 

### Study Limitations

We have some study limitations. First, the population and follow-up duration varied. Second, because we investigated the effect of CETP inhibitors on CVD outcomes, we only included studies with at least 100 participants and a 6-month follow-up. This limited our review to trials with minor, short-term lipid changes. Third, because TA-8995 is still being developed and no study has met our inclusion criteria, we were unable to include it in our analysis. Finally, dalcetrapib, anacetrapib, and evacetrapib all have varying effects on LDL-C and HDL-C and have diverse elimination half-lives; consequently, the CETP inhibitors may have had variable CVD benefits. Nevertheless, due to the low absolute number of adverse events, we found it reasonable to research this class of drugs as an assessment of the CVD effects of CETP inhibitors.

## 5. Conclusions

CETP inhibitors are associated with a reduced risk of MI and CVD mortality. No impact of CETP inhibitors was observed on MACEs, all-cause mortality, stroke, hospitalization due to acute coronary syndrome, and revascularization. Thus, there is still a need for additional and convenient lipid-lowering therapies, particularly for very high-risk patients. This information is imperative for clinicians to direct the right treatment for patients at risk, minimizing potential unnecessary interventional cardiology. 

## Figures and Tables

**Table 1:** Baseline characteristics of the included studies.

Study, Year	Drug Regimen	Arm Count		Age (Years)		Mean BMI		Men (%)		Statin Use ^‡^ (%)		HT (%)		Diabetes (%)		Mean LDL-C (mg/dL)		Mean HDL-C (mg/dL)	
		CETPi	Placebo	CETPi	Placebo	CETPi	Placebo	CETPi	Placebo	CETPi	Placebo	CETPi	Placebo	CETPi	Placebo	CETPi	Placebo	CETPi	Placebo
ACCELERATE, 2017 [29]	Evacetrapib	6038	6054	64.8	65	-	-	77	77	96.4	96.6	87.3	87.6	68.4	67.9	81.6	81.1	45.3	45.3
HPS3/TIMI55- REVEAL Collaborative Group, 2022 [27]	Anacetrapib	15,225	15,224	66	66	29	29	84	84	100	100	-	-	36	36	61	61	40	40
REVEAL, 2017 [26]	Anacetrapib	15,225	15,224	67	67	28.6	28.6	84	84	97.2	96.9	-	-	37.1	37.2	61	61	40	40
DEFINE, 2010 [10]	Anacetrapib	808	804	62.5	62.9	30.4	30.1	78	76	99	99	69	67	53	53	81.4	82.2	40.5	40.4
Ballantyne et al., 2017 [33]	Anacetrapib	290	292	60.3	60.9	27.7	27.8	76	69	100	100	64	72	9	6	87.2	88.7	43.5	43.6
Ballantyne et al., 2017 [34]	Anacetrapib	153	154	58.7	60.3	31.1	31	66	70	100	100	72	74	48	51	95.7	93	46.2	47.7
Teramoto et al., 2017 [35]	Anacetrapib	204	103	60.9	60.5	25.2	25.4	70	64	100	100	-	-	34	44	125.7	128.2	53.9	56
REALIZE, 2015 [36]	Anacetrapib	203	102	55	55.7	28.2	27.9	59	49	100	100	30	39	5	6	130	130	54	54
dal-OUTCOMES, 2012 [37]	Dalcetrapib	7938	7933	60.3	60.1	28.6	28.6	80	81	97	98	67	68	24	25	76.4	75.8	42.5	42.2
dal-VESSEL, 2012 [38]	Dalcetrapib	236	236	62.3	61.9	29.6	28.7	91	90	94	97	74	75	47	44	81.4	79.2	39.1	38.4
dal-PLAQUE, 2011 [39]	Dalcetrapib	63	65	62.6	64.6	29.6	29.8	80	83	81	92	73	73	30	30	73	73	42	46
Stein et al., 2009 [40]	Dalcetrapib	89	46	61.2	60.2	30.5	30.1	76	83	-	-	73	65	54	54	77	77	41	41

ACCELERATE: Assessment of Clinical Effects of Cholesteryl Ester Transfer Protein Inhibition with Evacetrapib in Patients at a High-Risk for Vascular Outcomes; BMI: body mass index; dal-OUTCOMES: Dalcetrapib on Cardiovascular Mortality and Morbidity in Clinically Stable Patients with a Recent Acute Coronary Syndrome; dal-PLAQUE: Safety and efficacy of dalcetrapib on atherosclerotic disease using novel non-invasive multimodality imaging; dal-VESSEL: A Study Assessing the Effect of Dalcetrapib on Vascular Function in Patients With Coronary Heart Disease (CHD) or CHD-Risk Equivalent Patients; DEFINE: Details of the Determining the Efficacy and Tolerability of CETP Inhibition with Anacetrapib; HDL-C: high-density lipoprotein cholesterol; HT: hypertension; LDL-C: low-density lipoprotein cholesterol; REALIZE: Anacetrapib as lipid-modifying therapy in patients with heterozygous familial hypercholesterolaemia; REVEAL: Randomized Evaluation of the Effects of Anacetrapib through Lipid Modification; TIMI: Thrombolysis In Myocardial Infarction. ^‡^ Amount of statins given along with CETPi medication.

## Data Availability

This meta-analysis adhered to the guidelines outlined in the Preferred Reporting Items for Systematic Reviews and Meta-Analyses (PRISMA) [31].

## Supplementary Materials

The following supporting information can be downloaded at: https://www.mdpi.com/article/10.3390/jcdd11050152/s1, Figure S1: Quality assessment of the independent researcher XY, [10,26,27,29,33,34,35,36,37,38,39,40,48]. Figure S2: Funnel plot, [10,27,29,33,35,36,37,38,40]. Figure S3: Forest plot of the outcome of stroke, [10,27,29,33,35,36,37,38,40]. Figure S4: Forest plot of the outcome of hospitalization due to acute coronary syndrome, [10,27,29,33,35,36,37,38,40]. Figure S5: Forest plot of the outcome of revascularization, [10,27,29,33,35,36,37,38,40]. Table S1: Search Strategy.

## Abbreviations

ACCELERATE	Assessment of Clinical Effects of Cholesteryl Ester Transfer Protein Inhibition with Evacetrapib in Patients at a High-Risk for Vascular Outcomes
ApoB	Apolipoprotein B
ASCVD	Atherosclerotic cardiovascular disease
BROADWAY	The Randomized Study to Evaluate the Effect of Obicetrapib on top of Maximum Tolerated Lipid-Modifying Therapies
BROOKLYN	Evaluate the Effect Of Obicetrapib in Patients with Heterozygous Familial Hypercholesterolemia on top of Maximum Tolerated Lipid-Modifying Therapies
CVD	Cardiovascular disease
CETP	Cholesteryl Ester Transfer Protein
Dal-OUTCOMES	Dalcetrapib on Cardiovascular Mortality and Morbidity in Clinically Stable Patients with a Recent Acute Coronary Syndrome
Dal-PLAQUE	Safety and efficacy of dalcetrapib on atherosclerotic disease using novel non-invasive multimodality imaging;
Dal-VESSEL	A Study Assessing the Effect of Dalcetrapib on Vascular Function in Patients with Coronary Heart Disease (CHD) or CHD-Risk Equivalent Patients
HDL	High-density lipoprotein
HDL-C	High-density lipoprotein cholesterol
HPS	Heart protection study
ILLUMINATE	Investigation of Lipid Level Management to Understand its Impact in Atherosclerotic Events
LDL	Low-density lipoprotein
LDL-C	Low-density lipoprotein cholesterol
MACE	Major adverse cardiovascular events
MI	Myocardial infarction
OCEAN	Randomized Study of Obicetrapib in Combination with Ezetimibe
PREVAIL	Cardiovascular Outcome Study to Evaluate the Effect Of Obicetrapib in Patients with Cardiovascular Disease
PRISMA	Preferred Reporting Items for Systematic Reviews and Meta-Analyses
RCTs	Randomized controlled trials
REALIZE	Anacetrapib as Lipid-Modifying Therapy in Patients with Heterozygous Familial Hypercholesterolaemia
REVEAL	Evaluation of the Effects of Anacetrapib Through Lipid Modification
ROSE	Randomized Study of Obicetrapib as an Adjunct to Statin Therapy
ROSE2	Study to Evaluate the Effect of Obicetrapib in Combination with Ezetimibe as an Adjunct to High-Intensity Statin Therapy
TIMI	Thrombolysis In Myocardial Infarction
TULIP	TA-8995 (obicetrapib): Its Use in Patients with Mild Dyslipidaemia
VLDL	Very low-density lipoprotein
