# Supplementary material for: Cholesteryl Ester Transfer Protein Inhibitors and Cardiovascular Outcomes: A Systematic Review and Meta-Analysis
Source: J Cardiovasc Dev Dis. 2024 May 16;11(5):152. doi: 10.3390/jcdd11050152 (PMC11122262; doi:10.3390/jcdd11050152)
Supplement: Supplementary file 1 [file jcdd-11-00152-s001.zip › jcdd-2892662-supplementary.pdf]

Supplementary Table S1: Search Strategy

Database	Query	Search details	Results
PubMed	CETP inhibitors OR anacetrapib OR evacetrapib OR dalcetrapib) AND Cardiovascular Outcomes	{("CETP"[All Fields] AND ("antagonists and inhibitors"[MeSH Subheading] OR ("antagonists"[All Fields] AND "inhibitors"[All Fields]) OR "antagonists and inhibitors"[All Fields] OR "inhibitors"[All Fields]} OR {("anacetrapib"[Supplementary Concept] OR "anacetrapib"[All Fields]) OR {"evacetrapib"[Supplementary Concept] OR "evacetrapib"[All Fields]) OR ("dalcetrapib"[Supplementary Concept] OR "dalcetrapib"[All Fields]) AND (("cardiovascular system"[MeSH Terms]) OR ("cardiovascular"[All Fields] AND "system"[All Fields]} OR "cardiovascular system"[All Fields] OR "cardiovascular"[All Fields] OR ("cardiovasculars"[All Fields]) AND ("outcome"[All Fields]) OR "outcomes"[All Fields])}	248
Embase	obicetrapib OR evacetrapib OR dalcetrapib OR anacetrapib OR cholesteryl ester transfer protein Inhibitor		245
Cochrane Library	obicetrapib OR evacetrapib OR dalcetrapib OR anacetrapib OR cholesteryl ester transfer protein Inhibitor		176
Medline	obicetrapib OR evacetrapib OR dalcetrapib OR anacetrapib OR cholesteryl ester transfer protein Inhibitor		312

Intention-to-treat	Unique ID	Study ID	Experimental	Comparator	Outcome	Weight	D1	D2	D3	D4	D5	Overall
	CVD1	Accelerate AM et al, 2017	NA	NA	NA	1	+	+	+	-	-	-
	CVD2	Ballantyne* et al, 2017	NA	NA	NA	1	+	+	+	-	+	-
	CVD3	Ballantyne et al, 2017	NA	NA	NA	1	+	+	+	+	-	-
	CVD4	DAL outcome et al, 2012	NA	NA	NA	1	+	+	+	+	+	+
	CVD5	DAL plaque ZA et al, 2011	NA	NA	NA	1	+	+	+	+	+	+
	CVD6	Dal vessel et al, 2012	NA	NA	NA	1	+	+	+	+	+	+
	CVD7	DEFINE CP et al, 2010	NA	NA	NA	1	+	+	+	-	+	-
	CVD8	E. Sammons et al, 2022	NA	NA	NA	1	+	+	+	-	+	-
	CVD9	Menon et al, 2020	NA	NA	NA	1	+	+	+	+	+	+
	CVD10	REALIZE JJ et al, 2015	NA	NA	NA	1	+	+	+	+	+	+
	CVD11	REVEAL et al, 2017	NA	NA	NA	1	+	+	+	+	+	+
	CVD12	Stein et al, 2010	NA	NA	NA	1	+	+	+	+	+	+
	CVD13	Teramoto et al, 2017	NA	NA	NA	1	+	+	+	+	+	+

+ Low risk
! Some concerns
- High risk

D1 Randomisation process
D2 Deviations from the intended interventions
D3 Missing outcome data
D4 Measurement of the outcome
D5 Selection of the reported result

Supplementary Figure S1: Quality assessment of the independent researcher XY. The majority of the studies were deemed to have a low risk of bias, but five of them were flagged for exhibiting a high risk of bias. This was primarily attributed to issues concerning how the outcome was measured and which outcomes were selected for reporting. Accelerate AM et al., 2017 [1]; Ballantyne* et al., 2017 Am J Cardiol Vol. 120 [2]; Ballantyne et al., 2017 [3]; DAL outcome et al., 2012 [4]; Dal plaque ZA et al., 2011 [5]; Dal vessel et al., 2012 [6]; DEFINE CP et al., 2010 [7]; E. Sammons et al., 2022 [8]; Menon et al., 2020 [9]; REALIZE JJ et al., 2015 [10]; REVEAL et al., 2017 [11]; Stein et al., 2010 [12]; Teramoto et al., 2017 [13];

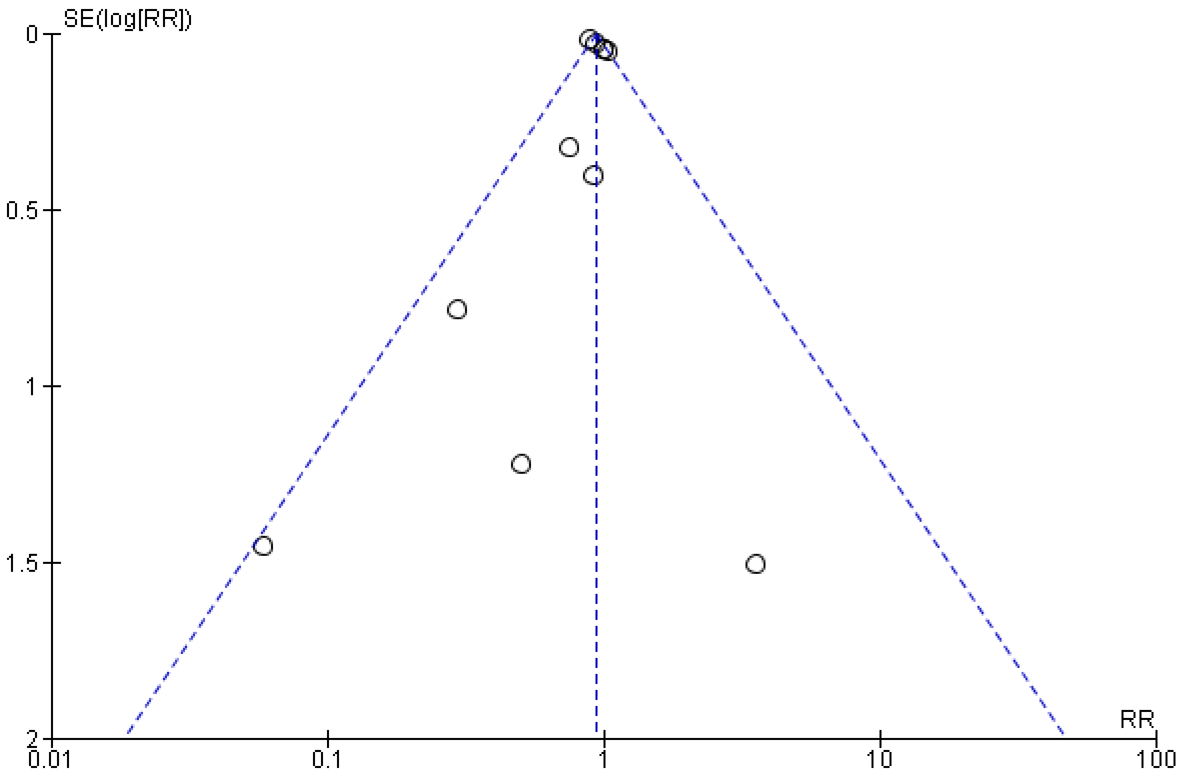

Supplementary Figure S2: Funnel Plot. An asymmetrical distribution of data points was noted in the funnel chart, indicating the possibility of publication bias within the studies. This pattern may also indicate a potential bias associated with smaller studies. Significance was determined by a p-value < 0.05, indicating statistical significance.

SUPPLEMENTARY FOREST PLOTS

Study or Subgroup	CETP Inhibitors Events	CETP Inhibitors Total	Placebo Events	Placebo Total	Weight	Risk Ratio M-H, Random, 95% CI
**1.4.1 Anacetrapib**						
Ballantyne* et al, 2017	0	290	2	292	0.1%	0.20 [0.01, 4.18]
DEFINE CP et al, 2010	5	803	5	804	0.8%	1.00 [0.29, 3.45]
E. Sammons et al, 2022	702	15224	754	15224	73.1%	0.93 [0.84, 1.03]
REALIZE JJ et al, 2015	0	203	0	102		Not estimable
Teramoto et al, 2017	0	204	0	103		Not estimable
**Subtotal (95% CI)**		**16724**		**16525**	**74.0%**	**0.93 [0.84, 1.03]**
Total events	707		761			
Heterogeneity: Tau² = 0.00; Chi² = 0.99, df = 2 (P = 0.61); I² = 0%						
Test for overall effect: Z = 1.42 (P = 0.15)						
**1.4.2 Dalcetrapib**						
DAL outcome et al, 2012	91	7938	73	7933	11.8%	1.25 [0.92, 1.69]
Dal vessel et al, 2012	0	236	0	236		Not estimable
Stein et al, 2010	0	89	0	46		Not estimable
**Subtotal (95% CI)**		**8263**		**8215**	**11.8%**	**1.25 [0.92, 1.69]**
Total events	91		73			
Heterogeneity: Not applicable						
Test for overall effect: Z = 1.41 (P = 0.16)						
**1.4.3 Evacetrapib**						
Accelerate AM et al, 2017	98	6038	98	6054	14.2%	1.00 [0.76, 1.32]
**Subtotal (95% CI)**		**6038**		**6054**	**14.2%**	**1.00 [0.76, 1.32]**
Total events	98		98			
Heterogeneity: Not applicable						
Test for overall effect: Z = 0.02 (P = 0.99)						
**Total (95% CI)**		**31025**		**30794**	**100.0%**	**0.97 [0.87, 1.08]**
Total events	896		932			
Heterogeneity: Tau² = 0.00; Chi² = 4.25, df = 4 (P = 0.37); I² = 6%						
Test for overall effect: Z = 0.50 (P = 0.61)						
Test for subgroup differences: Chi² = 3.26, df = 2 (P = 0.20), I² = 38.7%						

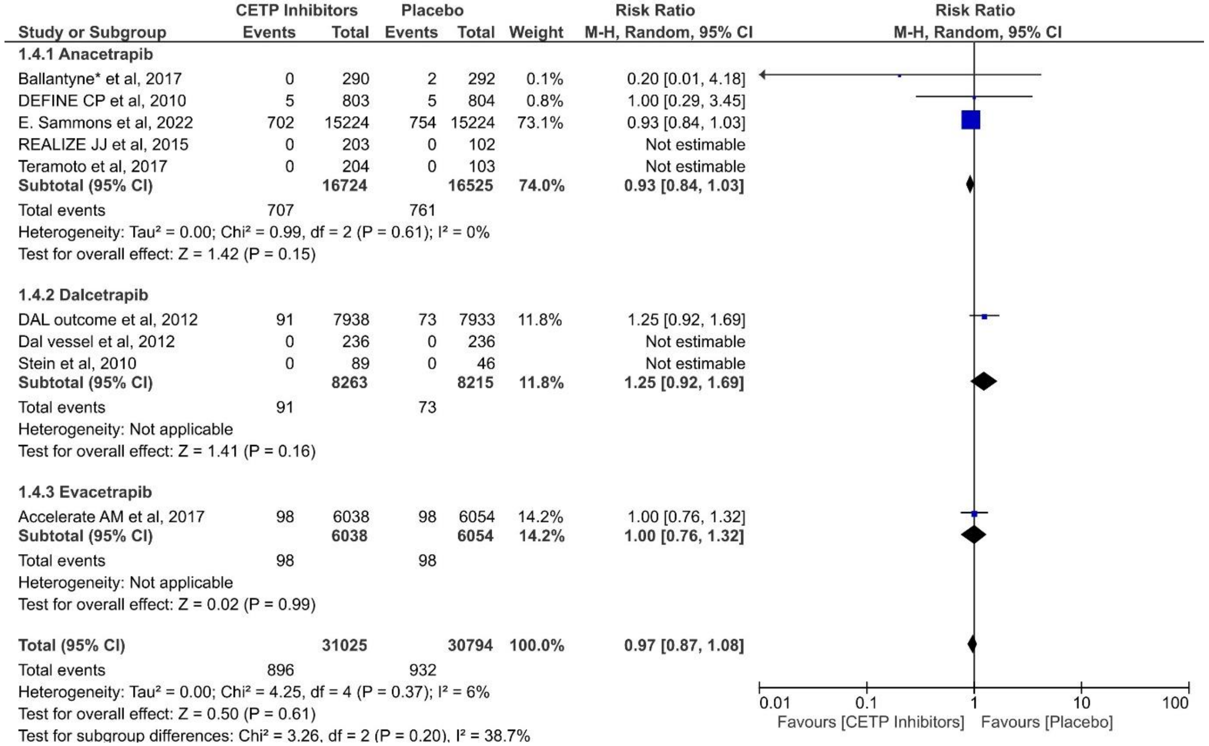

Supplementary Figure S3. Forest Plot of the Outcome of Stroke

Ballantyne* et al., 2017 Am J Cardiol Vol. 120 [2]; DEFINE CP et al., 2010 [7]; E. Sammons et al., 2022 [8]; REALIZE JJ et al., 2015 [10]; Teramoto et al., 2017 [13]; DAL outcome et al., 2012 [4]; Dal vessel et al., 2012 [6]; Stein et al., 2010 [12]; Accelerate AM et al., 2017 [1]. The values of each study (represented by black diamonds), in which the size is determined by 95% CI; the effect size of each individual study in the meta-analysis (represented by blue squares).

Study or Subgroup	CETP Inhibitors Events	CETP Inhibitors Total	Placebo Events	Placebo Total	Weight	Risk Ratio M-H, Random, 95% CI
**1.5.1 Anacetrapib**						
Ballantyne* et al, 2017	0	290	5	292	1.2%	0.09 [0.01, 1.65]
DEFINE CP et al, 2010	1	808	6	804	2.2%	0.17 [0.02, 1.37]
REALIZE JJ et al, 2015	3	203	0	102	1.1%	3.53 [0.18, 67.78]
Teramoto et al, 2017	0	204	0	103		Not estimable
**Subtotal (95% CI)**		**1505**		**1301**	**4.5%**	**0.34 [0.04, 2.60]**
Total events	4		11			
Heterogeneity: Tau² = 1.47; Chi² = 3.63, df = 2 (P = 0.16); I² = 45%						
Test for overall effect: Z = 1.04 (P = 0.30)						
**1.5.2 Dalcetrapib**						
DAL outcome et al, 2012	84	7938	92	7933	43.2%	0.91 [0.68, 1.22]
Dal vessel et al, 2012	0	236	1	236	1.0%	0.33 [0.01, 8.14]
Stein et al, 2010	0	89	0	46		Not estimable
**Subtotal (95% CI)**		**8263**		**8215**	**44.2%**	**0.90 [0.68, 1.21]**
Total events	84		93			
Heterogeneity: Tau² = 0.00; Chi² = 0.38, df = 1 (P = 0.54); I² = 0%						
Test for overall effect: Z = 0.67 (P = 0.50)						
**1.5.3 Evacetrapib**						
Accelerate AM et al, 2017	155	6038	146	6054	51.4%	1.06 [0.85, 1.33]
**Subtotal (95% CI)**		**6038**		**6054**	**51.4%**	**1.06 [0.85, 1.33]**
Total events	155		146			
Heterogeneity: Not applicable						
Test for overall effect: Z = 0.55 (P = 0.58)						
**Total (95% CI)**		**15806**		**15570**	**100.0%**	**0.93 [0.68, 1.28]**
Total events	243		250			
Heterogeneity: Tau² = 0.04; Chi² = 7.25, df = 5 (P = 0.20); I² = 31%						
Test for overall effect: Z = 0.44 (P = 0.66)						
Test for subgroup differences: Chi² = 1.83, df = 2 (P = 0.40), I² = 0%						

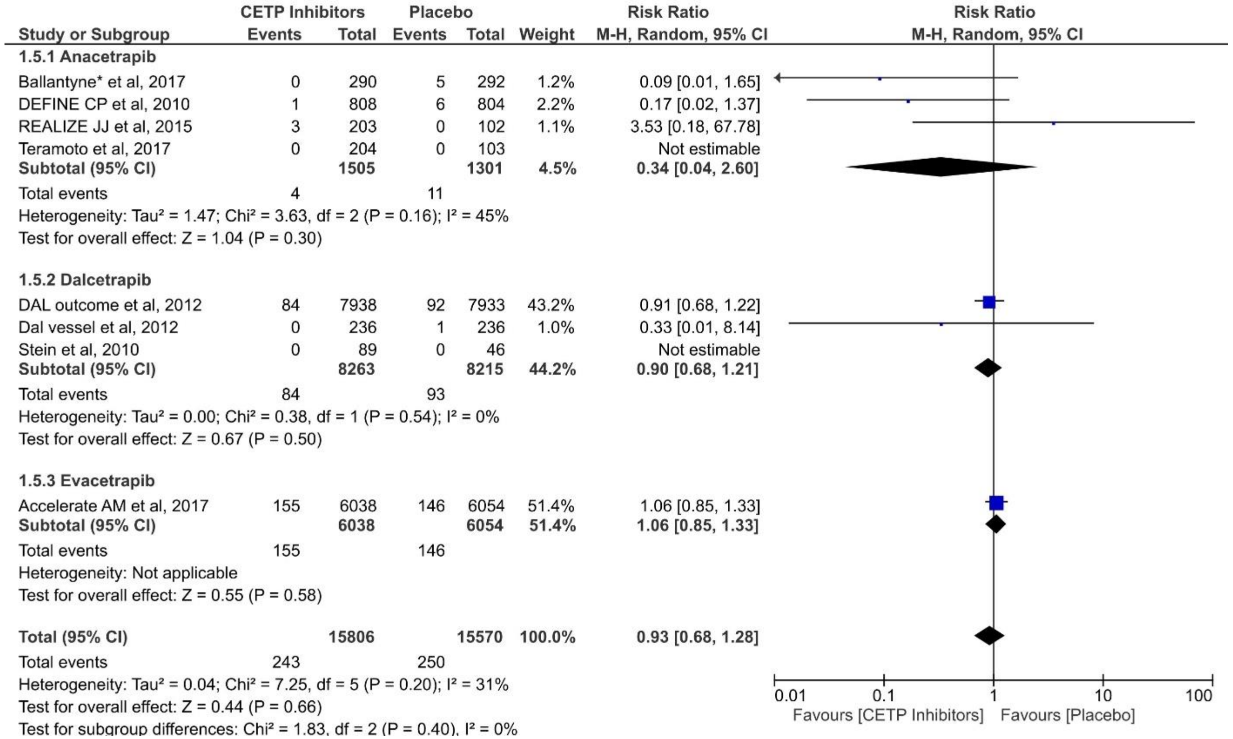

Supplementary Figure S4. Forest Plot of the Outcome of Hospitalization due to Acute Coronary Syndrome

Ballantyne* et al., 2017 Am J Cardiol Vol. 120 [2]; DEFINE CP et al., 2010 [7]; E. Sammons et al., 2022 [8]; REALIZE JJ et al., 2015 [10]; Teramoto et al., 2017 [13]; DAL outcome et al., 2012 [4]; Dal vessel et al., 2012 [6]; Stein et al., 2010 [12]; Accelerate AM et al., 2017 [1]. The values of each study (represented by black diamonds), in which the size is determined by 95% CI; the effect size of each individual study in the meta-analysis (represented by blue squares).

Study or Subgroup	CETP Inhibitors Events	CETP Inhibitors Total	Placebo Events	Placebo Total	Weight	Risk Ratio M-H, Random, 95% CI
**1.6.1 Anacetrapib**						
Ballantyne* et al, 2017	0	290	0	292		Not estimable
DEFINE CP et al, 2010	8	803	28	804	3.0%	0.29 [0.13, 0.62]
E. Sammons et al, 2022	1383	15225	1575	15224	34.5%	0.88 [0.82, 0.94]
REALIZE JJ et al, 2015	0	203	0	102		Not estimable
Teramoto et al, 2017	0	204	0	103		Not estimable
**Subtotal (95% CI)**		**16725**		**16525**	**37.4%**	**0.54 [0.18, 1.60]**
Total events	1391		1603			
Heterogeneity: Tau² = 0.55; Chi² = 7.90, df = 1 (P = 0.005); I² = 87%						
Test for overall effect: Z = 1.12 (P = 0.26)						
**1.6.2 Dalcetrapib**						
DAL outcome et al, 2012	674	7938	672	7933	31.3%	1.00 [0.90, 1.11]
Dal vessel et al, 2012	9	236	7	236	2.0%	1.29 [0.49, 3.40]
Stein et al, 2010	0	89	0	46		Not estimable
**Subtotal (95% CI)**		**8263**		**8215**	**33.2%**	**1.01 [0.91, 1.11]**
Total events	683		679			
Heterogeneity: Tau² = 0.00; Chi² = 0.25, df = 1 (P = 0.62); I² = 0%						
Test for overall effect: Z = 0.10 (P = 0.92)						
**1.6.3 Evacetrapib**						
Accelerate AM et al, 2017	487	6038	485	6054	29.4%	1.01 [0.89, 1.14]
**Subtotal (95% CI)**		**6038**		**6054**	**29.4%**	**1.01 [0.89, 1.14]**
Total events	487		485			
Heterogeneity: Not applicable						
Test for overall effect: Z = 0.11 (P = 0.91)						
**Total (95% CI)**		**31026**		**30794**	**100.0%**	**0.93 [0.81, 1.07]**
Total events	2561		2767			
Heterogeneity: Tau² = 0.01; Chi² = 15.62, df = 4 (P = 0.004); I² = 74%						
Test for overall effect: Z = 1.04 (P = 0.30)						
Test for subgroup differences: Chi² = 1.26, df = 2 (P = 0.53), I² = 0%						

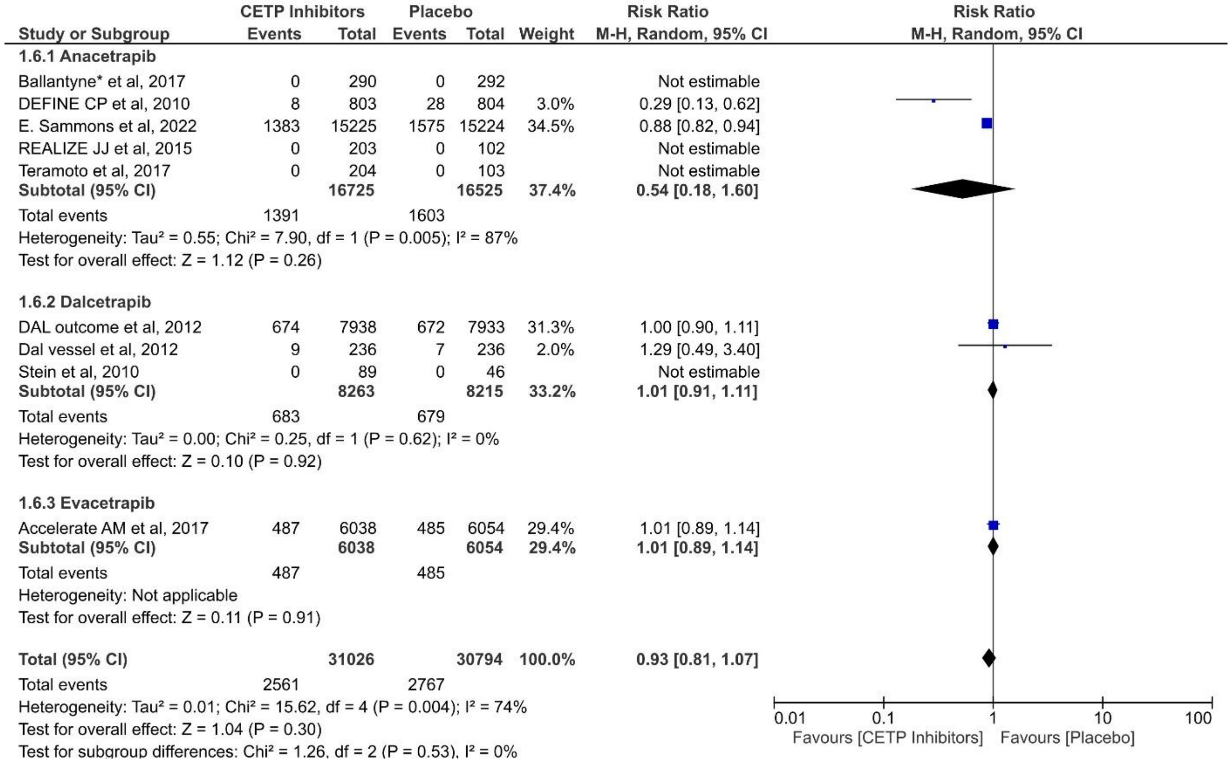

Supplementary Figure S5. Forest Plot of the Outcome of Revascularization

Ballantyne* et al., 2017 Am J Cardiol Vol. 120 [2]; DEFINE CP et al., 2010 [7]; E. Sammons et al., 2022 [8]; REALIZE JJ et al., 2015 [10]; Teramoto et al., 2017 [13]; DAL outcome et al., 2012 [4]; Dal vessel et al., 2012 [6]; Stein et al., 2010 [12]; Accelerate AM et al., 2017 [1]. The values of each study (represented by black diamonds), in which the size is determined by 95% CI; the effect size of each individual study in the meta-analysis (represented by blue squares).

References

[1]A. M. Lincoff, S. J. Nicholls, J. S. Riesmeyer, P. J. Barter, H. B. Brewer, K. A. A. Fox, C. M. Gibson, C. Granger, V. Menon, G. Montalescot, D. Rader, A. R. Tall, E. McErlean, K. Wolski, G. Ruotolo, B. Vangerow, G. Weerakkody, S. G. Goodman, D. Conde, D. K. McGuire, J. C. Nicolau, J. L. Leiva-Pons, Y. Pesant, W. Li, D. Kandath, S. Kouz, N. Tahirkheli, D. Mason, S. E. Nissen, and ACCELERATE Investigators, "Evacetrapib and Cardiovascular Outcomes in High-Risk Vascular Disease," *N. Engl. J. Med.*, vol. 376, no. 20, pp. 1933–1942, May 2017.

[2]C. M. Ballantyne, S. Shah, A. Sapre, T. B. Ashraf, S. C. Tobias, T. Sahin, P. Ye, Y. Dong, W. H.-H. Sheu, D.-H. Kang, P. R. Ferreira Rossi, Y. Moiseeva, I. R. Briones, A. O. Johnson-Levonas, and Y. B. Mitchel, "A Multiregional, Randomized Evaluation of the Lipid-Modifying Efficacy and Tolerability of Anacetrapib Added to Ongoing Statin Therapy in Patients With Hypercholesterolemia or Low High-Density Lipoprotein Cholesterol," *Am. J. Cardiol.*, vol. 120, no. 4, pp. 569–576, Aug. 2017.

[3]C. M. Ballantyne, S. Shah, U. Kher, J. A. Hunter, G. G. Gill, M. D. Cressman, T. B. Ashraf, A. O. Johnson-Levonas, and Y. B. Mitchel, "Lipid-Modifying Efficacy and Tolerability of Anacetrapib Added to Ongoing Statin Therapy in Patients with Hypercholesterolemia or Low High-Density Lipoprotein Cholesterol," *Am. J. Cardiol.*, vol. 119, no. 3, pp. 388–396, Feb. 2017.

[4]G. G. Schwartz, A. G. Olsson, M. Abt, C. M. Ballantyne, P. J. Barter, J. Brumm, B. R. Chaitman, I. M. Holme, D. Kallend, L. A. Leiter, E. Leitersdorf, J. J. V. McMurray, H. Mundl, S. J. Nicholls, P. K. Shah, J.-C. Tardif, R. S. Wright, and dal-OUTCOMES Investigators, "Effects of dalcetrapib in patients with a recent acute coronary syndrome," *N. Engl. J. Med.*, vol. 367, no. 22, pp. 2089–2099, Nov. 2012.

[5]Z. A. Fayad, V. Mani, M. Woodward, D. Kallend, M. Abt, T. Burgess, V. Fuster, C. M. Ballantyne, E. A. Stein, J.-C. Tardif, J. H. F. Rudd, M. E. Farkouh, A. Tawakol, and dal-PLAQUE Investigators, "Safety and efficacy of dalcetrapib on atherosclerotic disease using novel non-invasive multimodality imaging (dal-PLAQUE): a randomised clinical trial," *Lancet Lond. Engl.*, vol. 378, no. 9802, pp. 1547–1559, Oct. 2011.

[6]T. F. Lüscher, S. Taddei, J.-C. Kaski, J. W. Jukema, D. Kallend, T. Münzel, J. J. P. Kastelein, J. E. Deanfield, and dal-VESSEL Investigators, "Vascular effects and safety of dalcetrapib in patients with or at risk of coronary heart disease: the dal-VESSEL randomized clinical trial," *Eur. Heart J.*, vol. 33, no. 7, pp. 857–865, Apr. 2012.

[7]C. P. Cannon, S. Shah, H. M. Dansky, M. Davidson, E. A. Brinton, A. M. Gotto, M. Stepanavage, S. X. Liu, P. Gibbons, T. B. Ashraf, J. Zafarino, Y. Mitchel, and P. Barter, "Safety of Anacetrapib in Patients with or at High Risk for Coronary Heart Disease," *N. Engl. J. Med.*, vol. 363, no. 25, pp. 2406–2415, Dec. 2010.

[8]The HPS3/TIMI55-REVEAL Collaborative Group, Writing Committee, E. Sammons, J. C. Hopewell, F. Chen, W. Stevens, K. Wallendszus, E. Valdes-Marquez, R. Dayanandan, C. Knott, K. Murphy, E. Wincott, A. Baxter, R. Goodenough, M. Lay, M. Hill, S. Macdonnell, G. Fabbri, D. Lucci, M. Fajardo-Moser, S. Brenner, D. Hao, H. Zhang, J. Liu, B. Wuhan, S. Mosegaard, W. Herrington, C. Wanner, C. Angermann, G. Ertl, A. Maggioni, P. Barter, B. Mihaylova, Y. Mitchel, R. Blaustein, S. Goto, J. Tobert, P. DeLucca, Y. Chen, Z. Chen, A. Gray, R. Haynes, J. Armitage, C. Baigent, S. Wiviott, C. Cannon, E. Braunwald, R. Collins, L. Bowman, M. Landray, and REVEAL Collaborative Group, "Long-term safety and efficacy of anacetrapib in patients with atherosclerotic vascular disease," *Eur. Heart J.*, vol. 43, no. 14, pp. 1416–1424, Apr. 2022.

[9]V. Menon, A. Kumar, D. R. Patel, J. St John, J. Riesmeyer, G. Weerakkody, G. Ruotolo, K. E. Wolski, E. McErlean, P. C. Cremer, S. J. Nicholls, A. M. Lincoff, and S. E. Nissen, "Effect of CETP inhibition with evacetrapib in patients with diabetes mellitus enrolled in the ACCELERATE trial," *Bmj Open Diabetes Res. Care*, vol. 8, no. 1, p. e000943, Mar. 2020.
[10]J. J. P. Kastelein, J. Besseling, S. Shah, J. Bergeron, G. Langslet, G. K. Hovingh, N. Al-Saady, M. Koeijvoets, J. Hunter, A. O. Johnson-Levonas, J. Fable, A. Sapre, and Y. Mitchel, "Anacetrapib as lipid-modifying therapy in patients with heterozygous familial hypercholesterolaemia (REALIZE): a randomised, double-blind, placebo-controlled, phase 3 study," *Lancet Lond. Engl.*, vol. 385, no. 9983, pp. 2153–2161, May 2015.
[11]HPS3/TIMI55–REVEAL Collaborative Group, L. Bowman, J. C. Hopewell, F. Chen, K. Wallendszus, W. Stevens, R. Collins, S. D. Wiviott, C. P. Cannon, E. Braunwald, E. Sammons, and M. J. Landray, "Effects of Anacetrapib in Patients with Atherosclerotic Vascular Disease," *N. Engl. J. Med.*, vol. 377, no. 13, pp. 1217–1227, Sep. 2017.
[12]E. A. Stein, E. S. G. Stroes, G. Steiner, B. M. Buckley, A. M. Capponi, T. Burgess, E. J. Niesor, D. Kallend, and J. J. P. Kastelein, "Safety and tolerability of dalcetrapib," *Am. J. Cardiol.*, vol. 104, no. 1, pp. 82–91, Jul. 2009.
[13]T. Teramoto, H. Daida, K. Ikewaki, H. Arai, Y. Maeda, M. Nakagomi, M. Shirakawa, Y. Watanabe, T. Kakikawa, H. Numaguchi, A. O. Johnson-Levonas, and R. O. Blaustein, "Lipid-modifying efficacy and tolerability of anacetrapib added to ongoing statin therapy in Japanese patients with dyslipidemia," *Atherosclerosis*, vol. 261, pp. 69–77, Jun. 2017.
